# Insights into the structure and activity of prototype foamy virus RNase H

**DOI:** 10.1186/1742-4690-9-14

**Published:** 2012-02-10

**Authors:** Berit Leo, Maximilian J Hartl, Kristian Schweimer, Florian Mayr, Birgitta M Wöhrl

**Affiliations:** 1 Universität Bayreuth, Lehrstuhl Biopolymere, Universitätsstr. 30, D-95447 Bayreuth, Germany

**Keywords:** PFV, retroviral RNase H, C-helix, basic loop, NMR

## Abstract

**Background:**

RNase H is an endonuclease that hydrolyzes the RNA strand in RNA/DNA hybrids. Retroviral reverse transcriptases harbor a C-terminal RNase H domain whose activity is essential for viral replication. The RNase H degrades the viral genomic RNA after the first DNA strand is synthesized. Here, we report the biophysical and enzymatic properties of the RNase H domain of prototype foamy virus (PFV) as an independently purified protein. Sequence comparisons with other retroviral RNases H indicated that PFV RNase H harbors a basic protrusion, including a basic loop and the so-called C-helix, which was suggested to be important for activity and substrate binding and is absent in the RNase H domain of human immunodeficiency virus. So far, no structure of a retroviral RNase H containing a C-helix is available.

**Results:**

RNase H activity assays demonstrate that the PFV RNase H domain is active, although its activity is about 200-fold reduced as compared to the full length protease-reverse transcriptase enzyme. Fluorescence equilibrium titrations with an RNA/DNA substrate revealed a K_D _for the RNase H domain in the low micromolar range which is about 4000-fold higher than that of the full-length protease-reverse transcriptase enzyme. Analysis of the RNase H cleavage pattern using a [^32^P]-labeled substrate indicates that the independent RNase H domain cleaves the substrate non-specifically. The purified RNase H domain exhibits a well defined three-dimensional structure in solution which is stabilized in the presence of Mg^2+ ^ions.

**Conclusions:**

Our data demonstrate that the independent PFV RNase H domain is structured and active. The presence of the C-helix in PFV RNase H could be confirmed by assigning the protein backbone and calculating the chemical shift index using NMR spectroscopy.

## Background

Retroviral reverse transcription describes the formation of a double-stranded DNA using the single-stranded viral RNA genome as a template. The process is catalyzed by the viral reverse transcriptase which harbors a polymerase and an RNase H domain.

In most retroviruses reverse transcription takes place after the virus has entered the host cell. Spumaviruses, or foamy viruses (FVs), belong to a subfamily of the *retroviridae *and follow a distinct replication pattern unique among retroviruses: (a) reverse transcription occurs predominantly in the virus producing cell (b) the *pol*-gene coding for the viral enzymes is expressed from an independently spliced mRNA and (c) the viral protease is not cleaved off from the Pol precursor protein, leading to a mature protease-reverse transcriptase (PR-RT) [1-5]. Thus the mature PR-RT of FVs harbors a protease, polymerase and RNase H domain, each possessing a distinct enzymatic activity [4].

Retroviral RNases H are domains of the RT enzymes and degrade the RNA strand of the RNA/DNA hybrid which is formed in the first step of reverse transcription. This catalytic activity is essential during reverse transcription and leads to degradation of the genomic RNA during synthesis of the first or so-called minus DNA using the RNA as a template [6,7]. Mutations that inactivate the RNase H prevent viral replication [8,9].

RNase H cleavage by retroviral RTs, though generally not sequence specific, is also not devoid of any specificity. Retroviral RNases H exhibit three types of RNA cleavages: DNA 3' end directed, RNA 5' end directed, and internal. Specific cleavages are required during reverse transcription, when the extended tRNA and polypurine tract (PPT) primers, which are essential for the initiation of minus and plus strand DNA synthesis, have to be removed [10-17].

The isolated RNase H domain from human immunodeficiency virus type 1 (HIV-1) is enzymatically inactive [18-21], whereas that from Moloney murine leukemia virus (MoMLV) retains RNase H activity, albeit considerably lower than that of the full length RT [22-24]. Sequence alignments and structural comparisons revealed that MoMLV RNase H contains an additional helix-loop structure, also named the "basic protrusion", which includes the so-called C-helix and a downstream basic loop element [25-27]. This structural feature is also present in the human and *Escherichia coli *(*E. coli*) RNases H, both exist as independent proteins [28,29]. In contrast, the positively charged basic protrusion, which has been suggested to be important for substrate binding and/or activity [30-32], is missing in the HIV-1 RNase H domain, which is part of the RT enzyme [33]. Obviously, positively charged residues in the connection domain fulfill this function in HIV-1 RT [34]. Deletion of the putative C-helix of MoMLV RT results in replication defective viruses [30-32]. Similar to HIV-1 RNase H, the RNase H of the bacterium *Bacillus halodurans *is also devoid of a C-helix but still harbors a basic loop element. This RNase H carries an additional N-terminal extension, the so-called RNA-hybrid binding domain (RHBD), which is important for substrate binding [35].

Here, we analyzed the enzymatic and structural properties of the RNase H domain of prototype FV (PFV) because sequence alignments with other RNases H indicated the presence of a C-helix. We wanted to receive more information on this structural element, since HIV-1 RNase H does not possess a C-helix and the published structure of MoMLV RNase H could only be solved using a deletion mutant lacking the C-helix [25], whereas the crystal structure of the MoMLV RT in complex with duplex DNA (PDB: 1RW3) did not permit the building of a detailed model for the RNase H domain, since the electron density of this domain was not sufficiently well-ordered [36].

Activity assays and NMR spectroscopy of PFV RNase H show that it exhibits enzymatic activity and a well-defined three dimensional structure which harbors the basic protrusion including the C-helix.

## Results and Discussion

### Purification of the PFV RNase H domain

**Figure 1:**
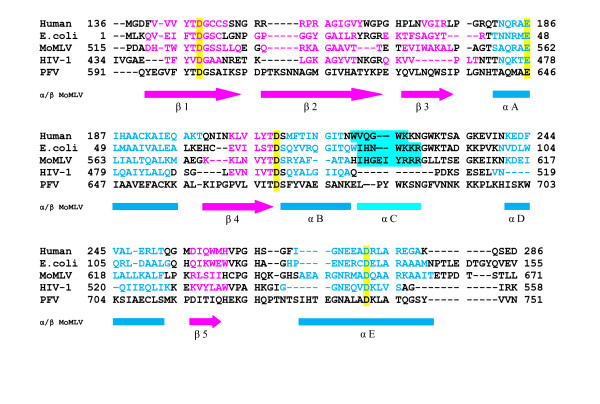
**Sequence alignment of different RNase H domains**. The RNases H from human origin, *E. coli*, MoMLV RT and HIV-1 RT are shown in comparison to the PFV RNase H. The numbers represent the amino acid numbers of the corresponding full length enzymes. The alignment was performed with the program CLC Protein Workbench 5 (version 5.5.2.0). Arrows below in pink indicate the β-strands in MoMLV RNase H, the corresponding amino acids in the other RNases H are highlighted by pink letters. Blue bars indicate α-helices in MoMLV RNase H, α-helical stretches of the other RNases H are highlighted by blue letters. The C-helices are highlighted by bright cyan boxes. The active site residues are highlighted in yellow.

Secondary structure predictions and sequence alignments of the PFV RNase H domain with the human and *E. coli *RNases H as well as with the retroviral RNases H from HIV-1 and MoMLV indicated the existence of a C-helix (Figure 1) in PFV and MoMLV RNases H, whereas the RNase H of HIV-1 does not possess a C-helix. The putative C-helix appears to be important for the activity of the free RNase H domain of MoMLV [25,30-32]. So far there is no structure of a retroviral RNase H domain available that actually shows the C-helix. Thus we wanted to prove experimentally by activity assays as well as NMR spectroscopy that PFV RNase H harbors a C-helix.

To analyze the properties of the RNase H domain of PFV we cloned the corresponding gene in an *E. coli *expression vector. A publication by Pfrepper *et al. *[37] suggested the existence of a cleavage site specific for FV protease (PR) between the polymerase and RNase H domains of FV RT at the sequence E^593^GVF^596^**↓**Y^597^TDG^600^. (The amino acid numbering always refers to the full length PFV PR-RT protein).

In contrast, we have shown recently that the only cleavage in the Pol precursor protein takes place between the RNase H and integrase domains. The mature PR-RT of PFV is monomeric, and the RNase H is not cleaved off [4,5]. Furthermore, secondary structure predictions indicated that the putative cleavage site suggested by Pfrepper *et al. *[37] is located in a β-strand (Figure 1). Cleavage in a secondary structure element can have detrimental effects on the structural integrity of the protein and thus this cleavage appears unlikely.

**Figure 2:**
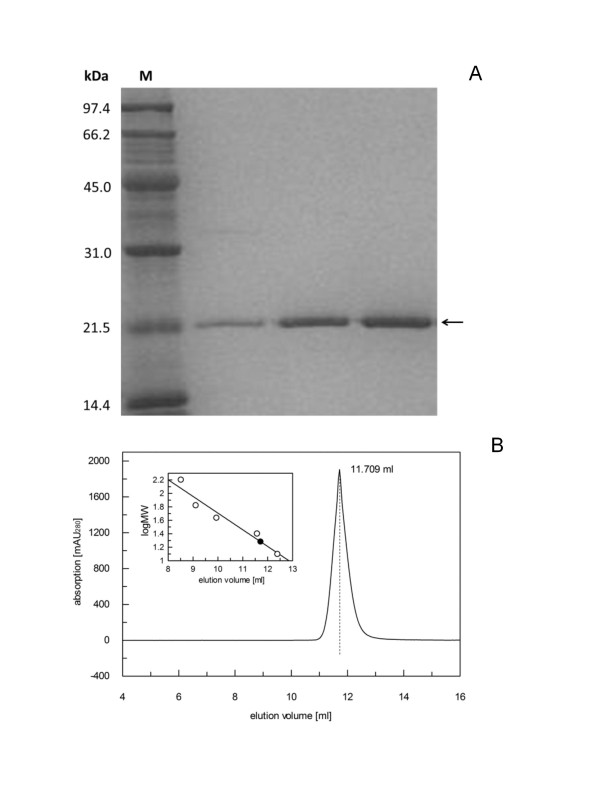
**Analysis of the purified PFV RNase H-(Q^591^-N^751^)**. **(A) ***SDS-PAGE of the purified PFV RNase H-(Q^591^-N^751^)*. PFV RNase H-(Q^591^-N^751^) was purified via Ni-affinity chromatography, TEV cleavage followed by a second Ni-affinity column to remove the 6His-GB1 tag. The flow through was concentrated and analyzed by a 19% SDS-PAGE and Coomassie staining. Lane M, molecular weight standard, the apparent molecular weights are indicated on the left. The arrow indicates the band of PFV RNase H-(Q^591^-N^751^) of which different amounts were loaded. **(B) ***Size exclusion chromatography of PFV RNase H-(Q^591^-N^751^) using an S75 HR 10/30 column*. The run was performed with 50 nmol of purified PFV RNase H-(Q^591^-N^751^) in 50 mM Na-phosphate buffer, pH 6.8, 100 mM NaCl and 0.5 mM DTT. The inset shows the best fit to the data obtained for the molecular masses of the standard proteins (open circles), which was used for the determination of the molecular mass of PFV RNase H-(Q^591^-N^751^) (closed circle).

To find out more about the structure and function of PFV RNase H, we expressed two different proteins, the first one starting with Y^597^TDG as suggested by Pfrepper *et al. *[37] as the N-terminal starting point of the PFV RNase H domain and the second one starting six amino acids further upstream with the sequence Q^591^YEGVFYTDG. This starting point was chosen, to avoid cleavage in the predicted β-strand (Figure 1). The two proteins were expressed with an N-terminal GB1 (immunoglobulin binding domain B1 of the streptococcal protein G) solubility tag. The resulting fusion proteins harbor a hexa-histidine tag at the N-terminus and a tobacco etch virus (TEV) protease cleavage site between GB1 and the corresponding RNase H domain. However, the short version of the RNase H (Y^597^-N^751^) proved unstable during expression and purification, indicating that the putative first β-strand of the RNase H is essential for the proper three-dimensional structure of the protein. Only the longer RNase H version, RNase H-(Q^591^-N^751^), could be purified successfully and was used for further analysis. After TEV cleavage and removal of the GB1 tag, the purity of the RNase H-(Q^591^-N^751^) was analyzed on a 19% SDS polyacrylamide gel (Figure 2A). To determine whether the RNase H is monomeric, we performed size exclusion chromatography of RNase H-(Q^591^-N^751^) (Figure 2B). The chromatogram shows a single peak corresponding to a molecular mass of 19 kDa (theoretical value: 18 kDa), demonstrating that the RNase H domain is monomeric.

### Enzyme activity

Mutational analysis performed with MoMLV RNase H indicated that the C-helix, which is part of the basic protrusion (L^681^-K^698 ^in PFV RNase H) is important for the activity of the separate RNase H domain [22-24,38]. The putative basic protrusion of PFV RNase H harbors five lysine residues (Figure 1).

**Figure 3:**
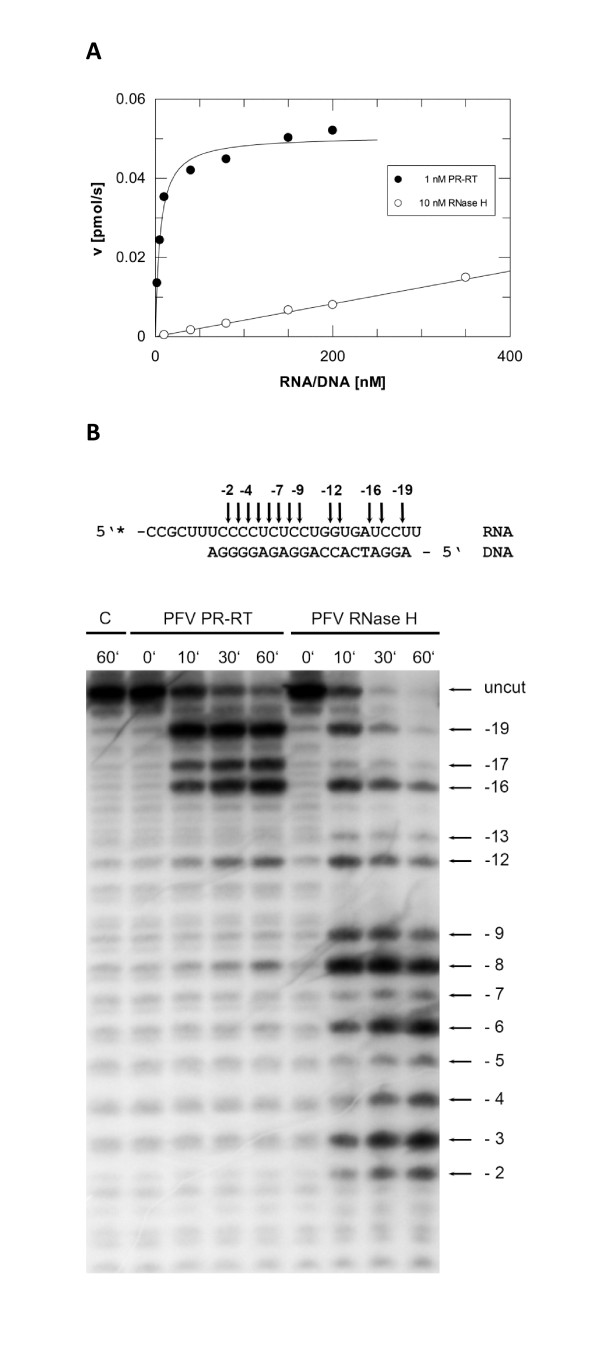
**RNase H activities of the full length PFV PR-RT and RNase H-(Q^591^-N^751^)**. **(A) ***Quantitative RNase H activity assay*. Steady state kinetic measurements of the velocity (pmol/s) of the RNase H activities were performed with 1 nM PR-RT (closed circles) and 10 nM RNase H-(Q^591^-N^751^) (open circles), respectively, using increasing amounts of a fluorescently labeled DNA/RNA hybrid substrate as indicated. For PFV PR-RT the following values were obtained: K_M _= 5.3 nM (± 0.8), v_max _= 0.05 pmol/s (± 0.002). The values were obtained by the fit program GraFit using the Michaelis-Menten equation v = v_max _[S]/(K_M _+ [S]) for the fitting procedure. The curve shows the best fit to the data. Due to its low activity, the kinetic parameters for the RNase H domain could not be determined. **(B) ***Qualitative RNase H assay*. The 20/27mer DNA/RNA primer/template substrate is shown on top of the figure. The cleavage sites are indicated by arrows in the sequence on top and at the corresponding band on the gel. The first nucleotide of the RNA hybridized to the 3'-OH nucleotide of the DNA strand is designated -1. 240 nM of the substrate were incubated with 30 nM PFV PR-RT or 20 μM of the RNase H domain for the times indicated on top of the gel. Reaction products were separated on a 15% sequencing gel and visualized by phosphoimaging.

Thus, we determined the cleavage activity of the free RNase H-(Q^591^-N^751^) in comparison with the RNase H domain of the full length PFV PR-RT. We chose a fluorescence assay for the detection of the activities and kinetic parameters since this assay proved to be very sensitive (Figure 3A). Our results indicate that the free PFV RNase H-(Q^591^-N^751^) domain is still active, although to a much lesser extent than the full length PR-RT. However, due to the low activity of PFV RNase H-(Q^591^-N^751^) we were only able to determine K_M _and v_max _values for the full length PFV PR-RT (K_M _= 5.3 nM (± 0.8), v_max _= 0.05 pmol/s (± 0.002)). Even when a 10-fold higher enzyme concentration of PFV RNase H-(Q^591^-N^751^) (Figure 3A) was used, the activities were more than 20 fold lower than for the PR-RT and saturation could not be achieved. Therefore, neither the fitting procedure nor graphical methods (Lineweaver-Burk, Eadie-Hofstee, Hanes) for K_M _and v_max _determination gave reliable results.

**Figure 4:**
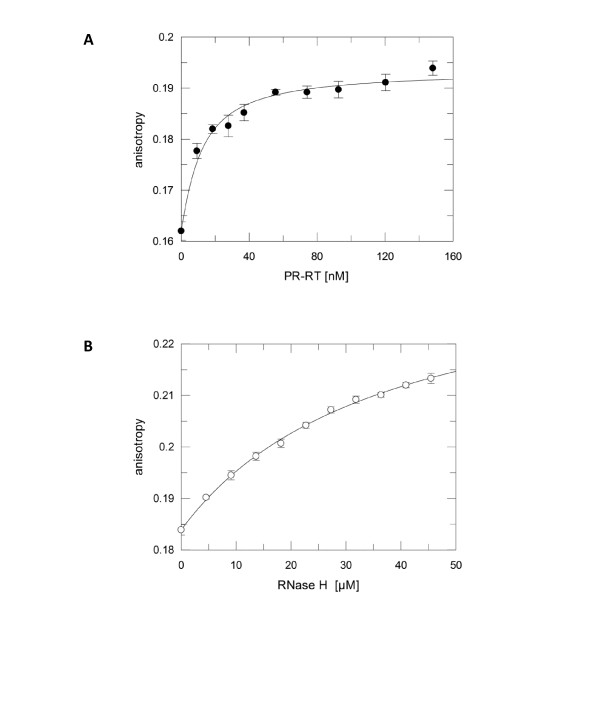
**Determination of K_D_-values by fluorescence anisotropy measurements**. 5 nM fluorescently labeled DNA/RNA primer/template substrate was titrated with PR-RT (closed circles) **(A) **or RNase H-(Q^591^-N^751^) (open circles) **(B) **as indicated. The fit of the curve to the data (equation 1) resulted in a K_D_-value of 9.1 nM (± 1.7) for the full length PFV PR-RT. The K_D_-value obtained for RNase H-(Q^591^-N^751^) is greater than 40 μM.

To further characterize the activity of RNase H-(Q^591^-N^751^), a qualitative RNase H assay was performed with a non-specific 20/27 mer DNA/RNA hybrid. The RNA was labeled with [^32^P] at the 5' end to visualize the cleavage products. To obtain cleavage of the substrate, we had to use almost 700 fold more RNase H-(Q^591^-N^751^) than PR-RT (Figure 3B). This confirms our results with the fluorescence assay described above, which also indicated a lower activity of RNase H-(Q^591^-N^751^) (Figure 3A). This is also reflected by the much higher dissociation constant (K_D_) determined (see below) for the RNase H domain (Figure 4). The cleavage pattern of the PR-RT confirms our previous data using a different substrate [4]. The cleavages performed by PR-RT are directed by the 3' end of the DNA primer, which binds to the active site of the polymerase domain [10]. Consequently, primary cleavage sites can be detected 16-19 nucleotides away from the 3' OH primer terminus of the hybrid. After 60 minutes, shorter cleavage products can be found up to position -8. In contrast, due to the lack of a polymerase active site, the cleavage pattern of the RNase H domain is different. After just 10 minutes, the RNase H domain exhibits a more equal distribution of cleavage sites spanning from -2 to -19. Nevertheless, certain sites appear to be preferred. Due to the high enzyme concentration used, we cannot exclude that this is derived from the binding of more than one enzyme molecule to the substrate. Our results are comparable to those obtained with the separate RNase H domain of MoMLV, which is also relatively non-specific even when specific substrates, i.e. containing the polypurine tract are used [23,24]

To get more information on the kinetic parameters, we set out to determine the dissociation constant (K_D_) for substrate binding. In the full length PR-RT enzyme, binding of the RNase H domain is amplified by the polymerase domain which also binds to the substrate and, due to its larger size, covers a larger portion of it. To analyze whether the higher RNase H activity of the full length PR-RT can,at least in part, be explained by a higher binding affinity of PR-RT, we performed fluorescence equilibrium titrations with a fluorescently labeled DNA/RNA primer/template substrate. The K_D _value of 9.1 nM (± 1.7) we obtained for the full length PFV PR-RT (Figure 4A) is comparable to previously published results [4]. However, again due to the fact that we were not able to reach saturation, the K_D_-value for the RNase H domain could not be determined precisely and is greater than 40 μM (Figure 4B) showing a binding affinity which is about 4000-fold lower than that of PFV PR-RT. These results imply a large contribution of the polymerase domain to substrate binding. Nevertheless, the K_D_-value gives a rough estimate of the K_M_-value, due to the fact that K_M _= (k_-1_/k_1_) + (k_2_/k_1_), or if k_2 _can be neglected K_M _= k_-1_/k_1 _= K_D_, indicating that the K_M _for the RNase H domain is greater than 40 μM.

### NMR analyses

**Figure 5:**
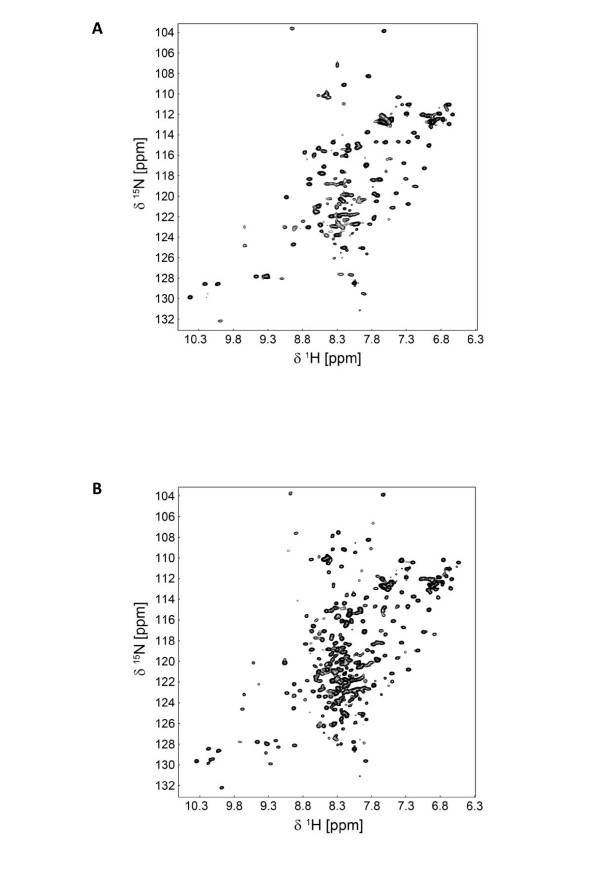
**Influence of Mg^2+ ^on the structure of PFV RNase H-(Q^591^-N^751^)**. ^1^H-^15^N-HSQC spectra of 45 μM PFV RNase H-(Q^591^-N^751^) were recorded at 298 K with a Bruker DRX 700 MHz spectrometer in 50 mM Na-phosphate pH 6.8, 100 mM NaCl, 0.5 mM DTT, 10% D_2_O in the absence **(A) **or presence **(B) **of 6 mM MgCl_2_.

Proper folding of the RNase H domain was analyzed by NMR spectroscopy using uniformly ^15^N-labeled protein. The activity of retroviral RNases H has been shown to be dependent on Mg^2+^, therefore NMR-spectra in the absence and presence of Mg^2+ ^ions were recorded (Figure 5). An NMR spectrum correlating resonance frequencies of amide protons and directly bonded ^15^N-labeled nitrogen atoms (2D [^1^H-^15^N] HSQC, *heteronuclear single quantum correlation*) allows the individual detection of peptide backbone amide signals. Thus, each signal represents a single amino acid of the peptide chain, and the spectrum serves as a fingerprint of the folding state of a protein. Adoption of a defined structure increases the dispersion of signals of amide protons in the HSQC spectrum to 6.9 - 9.7 ppm, relative to a random coil structure, which only exhibits an amide proton signal range from 7.8 - 8.7 ppm.

The spectra measured with the RNase H domain reveal that already in the absence of Mg^2+ ^well-dispersed signals (signal range: 6.9 - 9.7 ppm) typical for a protein with a defined tertiary structure are obtained (Figure 5A). However, the number of signals is not sufficient for a protein with 165 amino acids, and the signal intensities show significant differences. This is due to non-uniform NMR relaxation properties displaying a variety of dynamic processes not typical for a well-folded stable domain. In the presence of Mg^2+ ^the number of resonances increased remarkably, and the signal intensities show a more uniform distribution which is characteristic for a folded globular domain of 18 kDa (Figure 5B). These results indicate that for the structural integrity of the RNase H Mg^2+ ^is essential. It has been shown for other RNases H, that two Mg^2+ ^ions are bound in the catalytic center and are important for catalysis of the RNA cleavage by a two metal ion mechanism [29,39,40]. Although the presence of Mg^2+ ^is apparently not necessary for substrate binding by RTs [41-43], the Mg^2+ ^ions appear to stabilize the overall structure of the RNase H domain and make the protein more rigid since the Mg^2+ ^ions are coordinated by three highly conserved aspartate residues and one glutamate residue in RNases H of known structure. In the case of PFV RNase H the corresponding residues are D^599^, E^646^, D^669^, D^740 ^(Figure 1).

**Figure 6:**
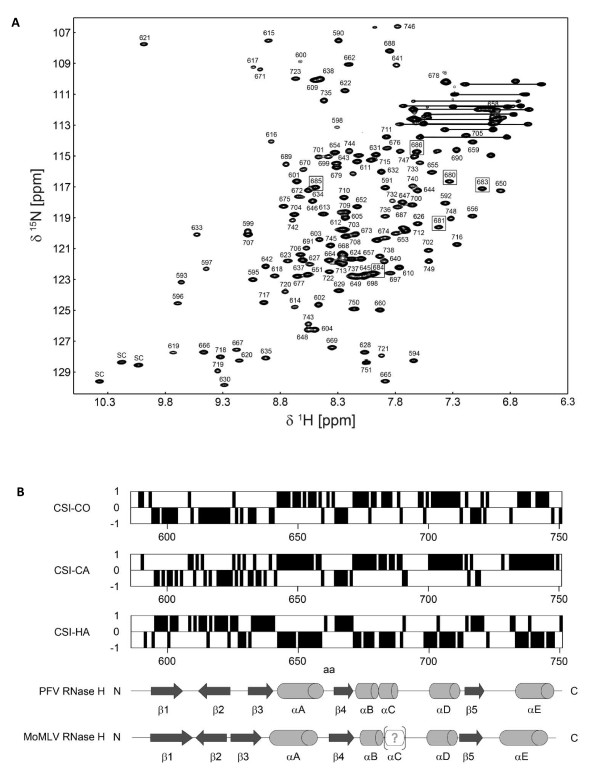
**NMR backbone assignment**. **(A) ***[^1^H, ^15^N] spectrum of the uniformly ^15^N,^13^C labeled PFV RNase H-(Q^591^-N^751^)*. Numbers refer to amino acid positions of the corresponding residues in the full length PFV PR-RT. NH2 side chains are connected by lines, the tryptophan side chains are marked by 'sc'. Several amino acids of the C-helix are marked by boxes. **(B) ***Secondary chemical shift indices for H_a_, C_a_, CO nuclei, and the consensus of PFV RNase H-(Q^591^-N^751^)*. Deduced β-strands are represented by arrows, and helical regions by cylinders. Precise amino acid numbering of the secondary structure elements are given in the text.

NMR data analysis of triple resonance experiments of uniformly ^15^N, ^13^C labeled RNase H-(Q^591^-N^751^) allowed the assignment of the backbone resonances for all residues except, D^607^, N^639^, N^692^-K^695^, Q^725^-N^728^, S^730^, I^731^, E^734^. The assigned [^1^H, ^15^N] HSQC spectrum is depicted in Figure 6A. Residues which constitute the C-helix (E^680 ^- S^686^), as determined by the chemical shift index (CSI) method (see below), are marked by boxes. The proline at position 682 is not visible in the HSQC spectrum due to the lack of a backbone N-H bond.

To determine the secondary structure elements and to prove the existence of the C-helix experimentally, we compared the observed NMR resonance frequencies with tabulated values found in random coil peptides by the CSI method (Figure 6B). The systematic deviations of backbone chemical shifts (resonance frequencies relative to reference standard) from random coil values in folded proteins form a valuable basis for the determination of sequence specific secondary structures and allow the experimental verification of postulated secondary structure elements. Helical secondary structures result in upfield chemical shift changes (lower resonance frequencies, lower ppm-values) of the α-protons (HA; negative CSI value) and downfield chemical shift changes (higher resonance frequencies, higher ppm-values) of the α- and carbonyl carbons (CA, CO; positive CSI value). β-strands exhibit the opposite direction of chemical shift changes compared to α-helical secondary structures.

In the RNase H domain, five sequence stretches (G^594^-I^603^, N^612^-Y^623^, N^631^-I^635^, V^664^-T^668^, I^716^-E^721^) show upfield chemical shifts of α- and carbonyl carbons and downfield chemical shifts of their α-protons, relative to random coil values and thus can be identified as β-strands (Figure 6B). The five sequence regions A^642^-L^658^, F^671^-K^679^, E^680^-S^686^, H^699^-S^711 ^and T^733^-G^746 ^exhibit the opposite direction of chemical shift changes compared to random coil values indicating the presence of α-helical secondary structures. The resulting overall sequence specific secondary structure resembles that of the RNase H domain of MoMLV (Figure 6B) and is consistent with other RNase H domains (Figure 1). The C-helix of PFV RNase H spans the α-helical region from E^680^-S^686 ^(Figure 6B).

In contrast, for MoMLV the C-helix was only predicted by sequence alignments and structure comparisons with other RNases H since no structure of the wild-type MoMLV RNase H could be obtained. The crystal structure of the MoMLV RNase H domain (PDB: 2HB5) could only be solved with a mutant protein lacking the C-helix [25]; thus no precise information on the orientation of the C-helix and its involvement in substrate binding could be made. Here we show that the solution structure analysis of PFV RNase H reveals the C-helix and adjacent regions. This is the first structural analysis of a retroviral RNase H where the C-helix can be detected.

## Conclusion

The detection of activity with the free PFV RNase H domain complements our NMR data which indicate that the protein is properly folded and harbors the basic protrusion including the C-helix. Our results suggest that similar to MoMLV the independent monomeric PFV RNase H domain is active due to the presence of the basic protrusion.

Furthermore, the data obtained here with PFV RNase H-(Q^591^-N^751^) together with previously published data [4,5] provide additional evidence that argues against the predicted PR cleavage between the polymerase and RNase H domain taking place [37].

These data expand the knowledge obtained with free MoMLV RNase H, which can also be expressed separately as an active enzyme, but with lower activity and less substrate specificity [22-24], and can now be used for further structural analyses by NMR since it has not been possible so far to obtain the structure of a retroviral RNase H domain harboring a C-helix.

## Methods

### Cloning, expression and protein purification

The gene coding for PFV RNase H starting either with the codon for Y^597 ^or Q^591 ^was amplified by PCR with a 5' primer including an *Nco*I site and a 3' primer with a *Bam*HI restriction site for further cloning. The fragment was cloned into the vector pET-GB1a (G. Stier, EMBL, Heidelberg, Germany) via *Nco*I and *Bam*HI. The expressed RNase H domains (ca. 18 kDa) were thereby fused to the 56 amino acids long B1 domain of *Streptococcal *protein G (GB1, 8740 Da). Both fusion proteins (ca. 27 kDa) harbor a hexa-histidine tag at the N-terminus and a tobacco etch virus (TEV) protease cleavage site between the GB1 and RNase H domains. Due to the *Nco*I cloning site, the cleaved RNase H variants habor an N-terminal extension of four additional amino acids (GAMG) upstream of the actual start of the RNase H amino acid sequence.

Gene expression was done in *E. coli *Rosetta DE3 (Novagen) in Luria broth [44] in the presence of 34 μg/ml chloramphenicol and 30 μg/ml kanamycin. For NMR studies M9 minimal medium [44] with 1× MEM vitamin solution (Gibco, Karlsruhe, Germany) supplemented with 1.5 g/l (^15^NH_4_)_2_SO_4 _(Cambridge Isotope Laboratories, Inc., Andover, MA, USA) and 4 g/l ^13^C-glucose (Euriso-Top, GIF-SUR-YVETTE, France) as the sole nitrogen and carbon sources were used. Cells were grown at 37°C to an optical density at 600 nm of 0.8. Expression was induced by adding 1 mM IPTG followed by incubation at 16°C over night. Cells were lysed using a Microfluidizer (MFTI Corporation, Newton, MA, USA) in a buffer containing 50 mM Na-phosphate pH 6.8, 1 M NaCl, 10 mM imidazole, 0.5 mM dithiothreitol (DTT).

The protein was purified by immobilized nickel affinity chromatography (5 ml HisTrap HP column, GE Healthcare, Munich, Germany) applying an imidazole step gradient for elution. Fractions containing GB1-RNase H were dialyzed against 50 mM Na-phosphate buffer pH 6.8, 300 mM NaCl, 0.5 mM DTT and cleaved with 1 mg TEV protease per 7 mg fusion protein for 21-24 h at 4°C. The TEV protease and the cleaved off GB1 tag were removed by a second Ni-affinity chromatography. The free PFV RNase H-(Q^591^-N^751^) was collected in the flow through. For HSQC-analysis in the presence of MgCl_2 _the purification was performed with 6 mM MgCl_2 _throughout the procedure.

Size exclusion chromatography of PFV RNase H-(Q^591^-N^751^) and purification of PFV PR-RT was performed as described previously [4,45].

### Quantitative RNase H activity assay

RNase H activity was measured quantitatively to determine steady state kinetic parameters by a fluorescence assay as described previously [4]. The RNA-strand of the hybrid was fluorescently labeled at the 3' end with 6-carboxy-fluorescein (6-FAM) (RNA-5'-CCGAUCGCUCUCCUGGUGAUCCUUUCC-6-FAM), whereas the DNA primer harbored a dabcyl quencher at the 5' end (DNA-5'-Dabcyl-GGAAAGGATCACCAGGAGAG) (biomers.net GmbH, Ulm Germany). To 1 nM of PFV PR-RT or 10 nM of PFV RNase H-(Q^591^-N^751^) increasing amounts of the RNA/DNA substrate were added as indicated (Figure 3A), in a buffer containing 50 mM Tris/HCl pH 8.0, 80 mM KCl, 6 mM MgCl_2 _and 0.5 mM DTT. An increase in fluorescence emission at 520 nm (excitation 495 nm) can be detected after cleavage of the RNA in the hybrid, which leads to dissociation of a small 6-FAM labeled RNA fragment from the dabcyl quencher. Fluorescence was measured on a Fluorolog-Tau-3 spectrofluorometer (HORIBA Jobin Yvon GmbH, Unterhaching, Germany). Initial cleavage rates were calculated using the linear slope of the reaction progress curve where less than 5% of substrate was cleaved. K_M _and v_max _values and their standard errors were obtained by fitting the data to the Michaelis-Menten equation using the fitting program GraFit 5.0 (Erithacus software).

### Qualitative RNase H assay

The RNA template (5'-CCGCUUUCCCCUCUCCUGGUGAUCCUU) (biomers.net GmbH, Ulm Germany) was purified on a denaturing 20% polyacrylamide/8 M urea gel. Ca. 30 pmol of the purified RNA was 5' end-labeled with 30 μCi γ[^32^P]-ATP (Hartmann Analytic, Braunschweig, Germany) and 20 U T4 polynucleotide kinase (New England Biolabs, Frankfurt, Germany) as described [43]. The DNA primer (5'-AGGATCACCAGGAGAGGGGA) (biomers.net GmbH, Ulm Germany) was hybridized to the RNA using a 1.2 fold molar excess of the DNA in 50 mM Tris/HCl, pH 7.0, 80 mM KCl by heating the sample to 90°C for 1 minute, followed by transfer to a heating block at 70°C and slow cooling to room temperature.

Qualitative RNase H reactions were performed at 25°C in a total volume of 25 μl in 50 mM Tris/HCl, pH 7.0, 80 mM KCl, 6 mM MgCl_2_, 0.5 mM DTT with 30 nM PR-RT or 20 μM RNase H-(Q^591^-N^751^), respectively. After preincubation of the sample for 5 min at 25°C, reactions were started by the addition of 240 nM RNA/DNA hybrid. Aliquots were taken after 10 min, 30 min and 60 min and stopped by the addition of an equal volume of urea loading buffer (50 mM EDTA, 0.1% xylene cyanol, 0.1% bromophenol blue, 8 M urea in 1 × TBE (Tris/Borate/EDTA)). Cleavage products were separated by denaturing gel electrophoresis using 15% polyacrylamide/8 M urea gels. After drying of the gel, the reaction products were visualized using a FLA 3000 phosphoimaging device (rayrest, Straubenhardt, Germany)

### Fluorescence anisotropy measurements

Fluorescence equilibrium titrations were performed with the full length PR-RT and the RNase H domain employing a Fluorolog-Tau-3 spectrofluorometer (HORIBA Jobin Yvon GmbH, Unterhaching, Germany) to determine the dissociation constant (K_D_) for an RNA/DNA template/primer substrate labeled with the fluorescent dye DY647 as described previously [43]. For the PR-RT a 24/40-mer DNA/RNA primer/template substrate (DNA-5'-ATCACCAGGAGAGGGGAAAGCGGA; RNA-5'-DY647-CUAAUUCCGCUUUCCCCUCUCCUGGUGAUCCUUUCCAUCC) (biomers.net GmbH, Ulm, Germany) was used as described [43]. However, for the RNase H domain a shorter 12/18-mer primer/template was chosen to avoid binding of more than one protein molecule to the hybrid region. The following sequences were used for the 12mer DNA primer 5'-ATCAGGTGTCGC and the fluorescently labeled 18mer RNA template 5'-DY647-AUAUAUGCGACACCUGAU (biomers.net GmbH, Ulm, Germany). Titrations were performed at 25°C in 1 ml of buffer consisting of 50 mM Tris/HCl pH 8.0, 80 mM KCl, 10 mM EDTA and 0.5 mM DTT. Upon excitation of the substrate at 652 nm, emission can be detected at 673 nm. The slit widths were set at 8 nm for PR-RT, and at 7 nm (excitation) and 6 nm (emission) for the RNase H-(Q^591^-N^751^) domain. 5 nM of substrate were used for PR-RT and 50 nM for the RNase H-(Q^591^-N^751^), respectively. Figure 4 shows representative titration experiments. After equilibration of the sample for 3 minutes, at least six data points with an integration time of 1 s were collected for each titration point. The standard deviation for each data point is represented by the error bars in Figure 4. K_D_-values and their standard errors were calculated by non-linear curve fitting of the anisotropy data to a two component binding equation (equation 1) using the software GraFit 5.0 (Erithacus software).

equation 1:

$$\text{A}=\frac{{\text{A}}_{\text{S}}+\text{P}\cdot \text{(R}\cdot {\text{A}}_{\text{ES}}\cdot {\text{A}}_{\text{S}}\text{)}}{1-\text{P}+\text{R}\cdot \text{P}}$$

with

$$P=\frac{\left[ES\right]}{{\left[S\right]}_{0}}=\frac{K_{D}+{\left[E\right]}_{0}+{\left[S\right]}_{0}-\sqrt{{\left(K_{D}+{\left[E\right]}_{0}+{\left[S\right]}_{0}\right)}^{2}-4\cdot {\left[S\right]}_{0}\cdot {\left[E\right]}_{0}}}{2\cdot {\left[S\right]}_{0}}$$

where A = anisotropy measured; A_S _= anisotropy of unbound substrate DNA/RNA; A_ES _= anisotropy of the complex; [ES] = concentration of enzyme/substrate complex; [E]_0 _= total concentration of enzyme (PR-RT or RNase H-(Q^591^-N^751^); [S]_0 _= total concentration of substrate DNA/RNA; K_D _= dissociation constant; R = ratio of fluorescence intensities of the bound and free substrate.

### NMR analyses

1 mM uniformly ^15^N labeled RNase H-(Q^591^-N^751^) was analyzed in 5 mM Na-phosphate pH 6.8 or 7.0, 100 mM NaCl, 0.5 mM DTT, 10% D_2_O (v/v) in the absence or presence of 6 mM MgCl_2_. For NMR resonance assignment standard double and triple resonance through-bond correlation experiments [46,47] were recorded using 1 mM ^15^N,^13^C labeled RNase H-(Q^591^-N^751^), in 5 mM Na-phosphate pH 7.0, 100 mM NaCl, 6 mM MgCl_2_, 0.5 mM DTT, 10% D_2_O (v/v) at 25°C on a Bruker Avance 700 MHz spectrometer equipped with a cryogenically cooled probe. In-house protocols were used to process the NMR data and the program NMRview were utilized for analysis (B.A. Johnson, Merck, Whitehouse Station, NJ, USA).

### Data deposition

The PFV RNase H-(Q^591^-N^751^) assignments have been deposited in the BioMagResBank, accession number: 17745

## Abbreviations

E. coli: *Escherichia coli*; FV: foamy virus; GB1: immunoglobulin binding domain B1 of streptococcal protein G; HIV-1: human immunodeficiency virus type 1; IN: integrase; IPTG: isopropyl-thiogalactoside; PFV: prototype foamy virus; MoMLV: Moloney murine leukemia virus; PPT: polypurine tract; PR: protease; RHBD: RNA-hybrid binding domain; RNase H: ribonuclease H; RT: reverse transcriptase; TEV: tobacco etch virus;

## Competing interests

The authors declare that they have no competing interests.

## Authors' contributions

BMW conceived and coordinated the study. BL and FM performed the experiments. BL, BMW, MJH and KS analyzed the data. BMW, BL and KS wrote the paper. All authors read and approved the manuscript.
